# Clinical and ultrastructural study after partial inferior turbinectomy

**DOI:** 10.1016/S1808-8694(15)31016-8

**Published:** 2015-10-19

**Authors:** Rogério Dutra Bandos, Valder Rodrigues de Mello, Maria Dolores Seabra Ferreira, Maria Rossato, Wilma Terezinha Anselmo-Lima

**Affiliations:** a Master's Degree (free lance professional).; b M.D., Professor Doctor.; c Laboratory Technician.; d Laboratory Technician.; e Assistant Professor. Ribeirão Preto Medical School - University of São Paulo.

**Keywords:** chronic nasal obstruction, inferior turbinate mucosa, ultrastructural changes on the mucosa, partial inferior turbinectomy, epithelial alterations

## Abstract

We report clinical and histological results obtained after partial inferior turbinectomy (PIT), surgery indicated for the treatment of chronic nasal obstruction.

**Methods:**

Twenty patients were divided into two groups submitted to PIT plus septoplasty and PIT alone. The patients were reassessed clinically and histologically by means of a biopsy of the regenerated areas in the inferior turbinates at two different times after PIT, i.e., after 8 to 12 months (group A) and after two years (group B).

**Results:**

The clinical results proved to be satisfactory for the relief of nasal obstruction in group A and unsatisfactory in group B. However, better histological results with better recovery and epithelial differentiation of the regenerated mucosa of the inferior turbinates after PIT were observed in group B.

**Conclusion:**

Surgery proved to be effective on a short-term but not on a long-term basis, and histological recovery did not accompany improvement of clinical signs and symptoms.

## INTRODUCTION

Breathing well is a condition directly related to quality of life. A good breathing demands good permeability of the nasal airways, the physiological entry door of air flow. Chronic nasal obstruction is a symptom responsible for most patients’ visit to otorhinolaryngologists in their daily practices. The evolution in Rhinology currently offers a wide array of options that allows for a better understanding and treatment of pathologies related to nasal obstruction, through new diagnostic and therapeutic weaponry. However, many times, such a task still poses a challenge. Turbinate hypertrophy and nasal septal deviation are the main causes of nasal obstruction. The association of septal deviation with inferior turbinate hypertrophy on the opposite side of the deviated septum, in a vicarious way, is a frequent finding in nose exams. Modern pharmacology offers a large number of options for clinical treatment of nasal obstruction due to turbinate hypertrophy, whatever the origin it may have (allergic, idiopathic, drug-related or others), and by immunology, in allergy cases. However, despite the fact of it still being a controversial issue, most authors agree that when clinical treatment is not enough to offer good nasal permeability, surgical treatment should be indicated^1^, ^2^. Today, inferior turbinate hypertrophy can be corrected with total, partial or submucosal turbinectomies, and turbinoplasties, besides other procedures, such as electrocautery, cryosurgery, laser vaporization and new technologies, such as, somnoplasty or coblation^3^.

Partial inferior turbinectomy (PIT) is a relatively simple surgical technique, used at the Otorhinolaryngology Department at Hospital das Clínicas at Riberão Preto Medical School - USP (HC-FMRP) for more than 15 years. This study aims to: 1- contribute to the understanding of PIT clinical benefits offered to patients with chronic nasal obstruction caused by inferior turbinate hypertrophy, through the analysis of a group of patients on the earlier post-operative stage (8 months to one year), and another group on a later stage (after two years); 2- describe the ultra structural findings on the regenerated inferior turbinate mucosa after PIT, with emphasis on ciliary recovery; 3- establish a relationship between the clinical situation of these patients and the ultra structural findings on the re-epithelized inferior turbinate mucosa.

## MATERIALS AND METHODS

The twenty patients selected were divided into two groups with ten patients per group.

Eleven females and nine males. The patients’ ages on the date of reassessment varied from 12 to 57 years, with an average of 25 in group A and 23 in group B. The inclusion criteria for patients on the study were chronic nasal obstruction without response to clinical treatment, inferior turbinate hypertrophy (uni or bilateral, allergic or not), with or without septal deviation as causal agents. All patients were submitted to uni or bilateral PIT. Thirteen patients were also submitted to septoplasty (Killian or Cottle techniques), and the surgical result being considered satisfactory (centered septum or without significant deviations). PIT was preceded by adrenalin solution injection (concentration 1:100.000), followed by a medial dislocation of turbinates. The amount of excised tissue varied according to the degree of hypertrophy of the turbinate, with the removal of soft tissue (mucosa and lamina propria) and bone of the adjacent turbinate, basically all along the anterior-posterior extension of the free border. An angled scissors was used for excision. Exclusion criteria for candidates were: presentation of other diseases related to nasal obstruction, such as nasal polyposis, infectious rhinosinusitis detected at some point during assessments, alterations of nasal valve, hypertrophy of middle turbinate or bubble-like middle turbinate, cystic fibrosis or primary ciliary diskinesia, and also patients with tuberculosis, chronic renal disease, diabetes or immunodeficiency. Those patients using sympathomimetic vasoconstrictors in an abusive manner did not participate due to its possible effects on nasal mucosa. Also those patients with an incomplete pre-operative clinical assessment were excluded and those who missed the first postoperative return visits, with inadequate biopsy material for histological study or those who refused to take part in the study.

Group A: Ten patients, reassessed clinically and histologically (nasal biopsy), between eight and twelve months after PIT (10 months average) were considered short term post-operative.

Group B: Ten patients, reassessed clinically and histologically two years after PTT (25 months average) were considered medium term postoperative. On this assessment, patients were submitted to a new interview and complete otorhinolaryngological exam. The place chose for biopsy was between 2 to 3 cm posterior to the beginning of the anterior end of the operated inferior turbinate, on its medial face. In the bilateral PIT cases, we agreed to biopsy the left side.

The present study was approved by the HC-FMRP Medical Ethics Committed under protocol # 1000. Once removed, a sample of the inferior turbinate went through a set of stages in order to be prepared to undergo electron microscopy. Immersion on a bottle with glutaraldehyde fixation solution at 3% in phosphate buffer, kept in a thermal container at 4 degrees Celsius during two hours. Washed in phosphate buffer. Post-fixation with osmium tetroxide at 1%. Dehydratation on acetone in increasing concentration (30, 50, 70, 90 and 95%), changing the concentration at every 10 minutes, and in three sessions of 20 minutes for the 100% concentration. Araldite infiltration 6.005 with propylene oxide at 1:1 ratio during 48 hours. Later on, the sample was included into the same pure resin for 72 hours at 60 degrees Celsius. The blocks obtained were trimmed and cut on an ultra microtome, and semi-fine cuts were obtained (0.5 micrometer) for study on optical microscopes. On this stage, the cuts were set up on slides, stained with toluidine blue 2%, on pH 12.0, in order to select the areas with adequate orientation for the ultra-fine cuts (60-70 nanometers). These were extended in copper bars, contrasted with uranyl acetate at 4% for 15 minutes and lead citrate 0.3% during 15 more minutes, and washed again. Finally, the material was examined and electromicrographed with a Phillips 208 transmission electron microscope. A histological analysis of biopsies was done by the same histopathologist.

## RESULTS

### Pre-operative results

**Figure 1:**
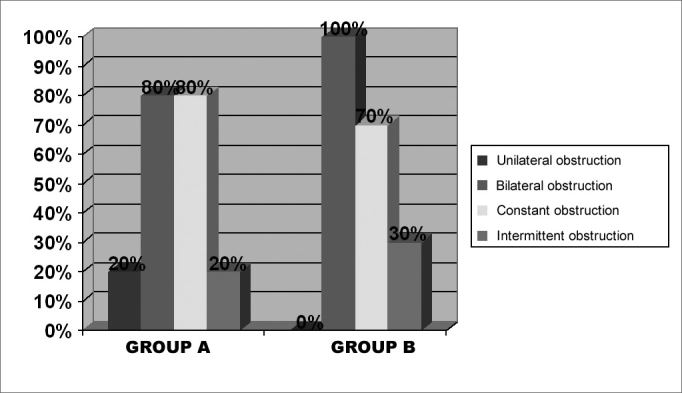
Percentage graph of nasal obstruction features on groups A and B, regarding its laterality and duration.

**Figure 2:**
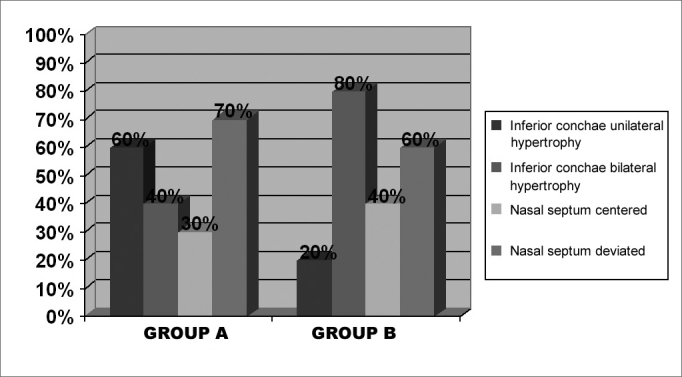
Percentage graph of the findings on the physical exam of groups A and B patients, regarding laterality of inferior turbinate hypertrophy and septal deviation.

**Figure 3:**
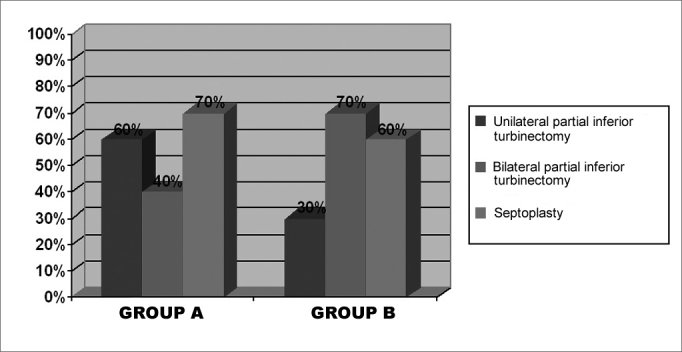
Graphic representation of percentages of surgical procedures done on groups A and B patients (N=10 each group).

On the pre-operative stage, we chose interview and physical exam data from the twenty patients included in this study, ten from each group. Figure 1 shows the statistical data (percentage) of nasal obstruction features, according to the laterality and duration of symptoms. Table 1 refers to the main symptoms that may be related to chronic nasal obstruction: itching, secretion, sneezing, hyposmia, oral breathing, headaches and subjective cacosmia, showing the number of patients affected in both groups. One patient from each group reported having snored as well. The prevalence of uni or bilateral inferior turbinate hypertrophy, and the presence or the absence of septal deviation detected at the physical exam (anterior and posterior rhinoscopy), are on Figure 2. Some patients used up to three different types of drugs to deal with nasal obstruction, and still had the indication for surgical intervention. It is worth saying that most patients reported to use prescribed drugs regularly. Figure 3 depicts the surgical procedures adopted for each group, with the statistical analysis.

**Table 1:** Frequency of pre-operative symptoms on groups A and B patients.

Symptoms	Group A	Group B
	N %	N %
Itch	7 70	3 30
Secretion	6 60	3 30
Sneezing	5 50	5 50
Hyposmia	8 80	4 40
Oral breathing	10 100	10 100
Headache	0 0	1 10
Cacosmia	3 30	1 10

### Post-operative results

**Figure 4:**
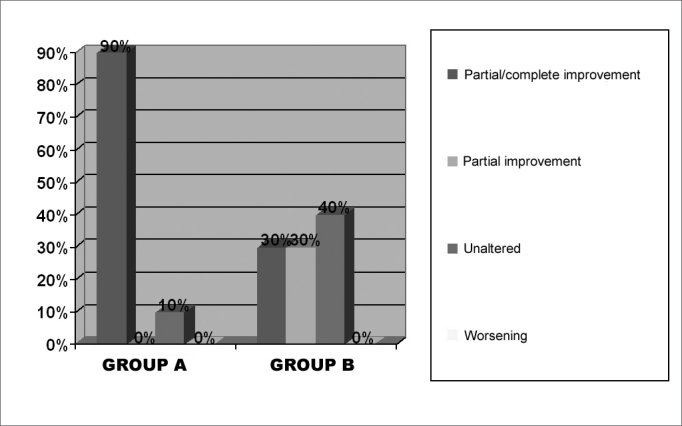
Percentage graph of the post-operative evolution regarding nasal obstruction on the groups.

**Figure 5:**
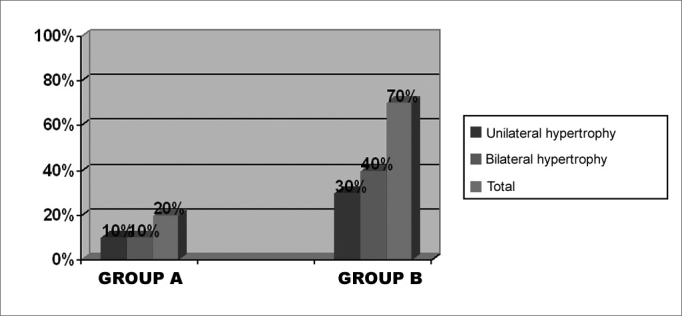
Percentage frequency of physical exam results on the postoperative of the groups.

**Figure 6:**
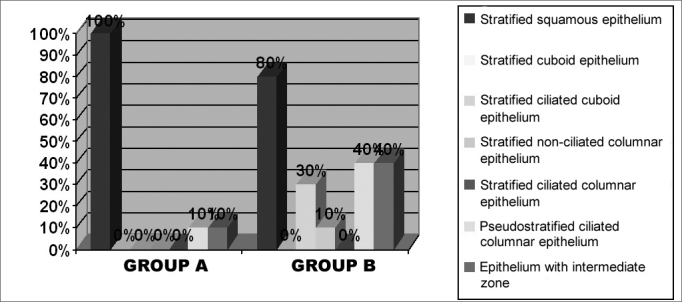
Frequency of different types of epithelium found in the groups.

**Figure 7:**
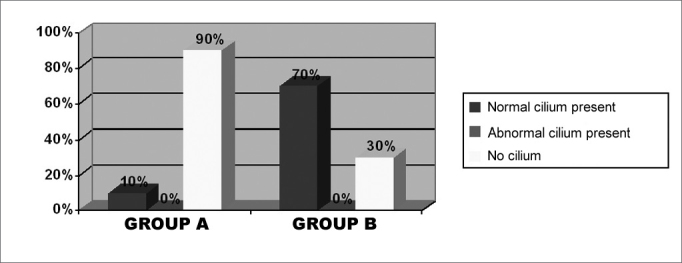
Frequency of ciliary features found on patients of both groups.

The clinical and histological results obtained on the post-operative stage are showed separately on groups A (short-term post-operative) and B (medium term post-operative). Table 2 corresponds to post-operative symptom findings, on groups A and B respectively, according to the patients’ report on total or subtotal improvement (which were considered as good results), partial improvement, unaltered or worsening of symptoms. Nasal obstruction, the most important symptom, is represented graphically (Figure 4). We did not observe post-operative complications, such as significant bleeding or infection. Figure 5 shows the physical exam findings (anterior and posterior rhinoscopy), in groups A and B, regarding mucosa trophyc features on the inferior turbinates. Alterations such as synechiae, polyps, rhinosinusitis, and nasal turbinate degeneration were not found. It is worth mentioning that the macroscopic aspect of the operated turbinates was very similar to those turbinates never operated. According to what it was established on the inclusion criteria for this study, the nasal septum in all patients (submitted or not submitted to septoplasty), on the post-operative stage, was centered or without a significant deviation. The frequency of the different epithelial types observed on postoperative biopsies, was listed on Figure 6, organized according to the differentiation degree of the epithelium (from less to more differentiation). In some samples, it was noticed up to two different epithelial types, with transition areas between two epithelia, that is why the total number of epithelial types described in each group is higher than 10. This type of classification can be easily observed on light microscopy. The ultra structural features of the cilia were observed on electronic microscopy, where we could verify that, when they are present, the cilium appears to be normal in its ultra structure (Figure 7). We did not observe any ciliary ultra structural alteration, such as short, composed or cilium with tubular alterations.

**Table 2:** Post-operative evolution of symptoms on groups A and B patients.

Symptom	Total/Subtotal Improvement		Partial Improvement		Unaltered		Worsening	
	Group A	Group B	Group A	Group B	Group A	Group B	Group A	Group B
Nasal obstruction	9	3	0	3	1	4	0	0
Itch	3	1	2	2	5	7	0	0
Secretion	2	0	2	2	6	8	0	0
Sneezing	3	2	2	1	5	7	0	0
Hyposmia	7	1	0	1	3	7	0	1
Oral breathing	7	3	2	3	1	4	0	0
Headache	0	1	0	0	10	9	0	0
Cacosmia	2	0	0	0	8	9	0	1

**Figure 8:**
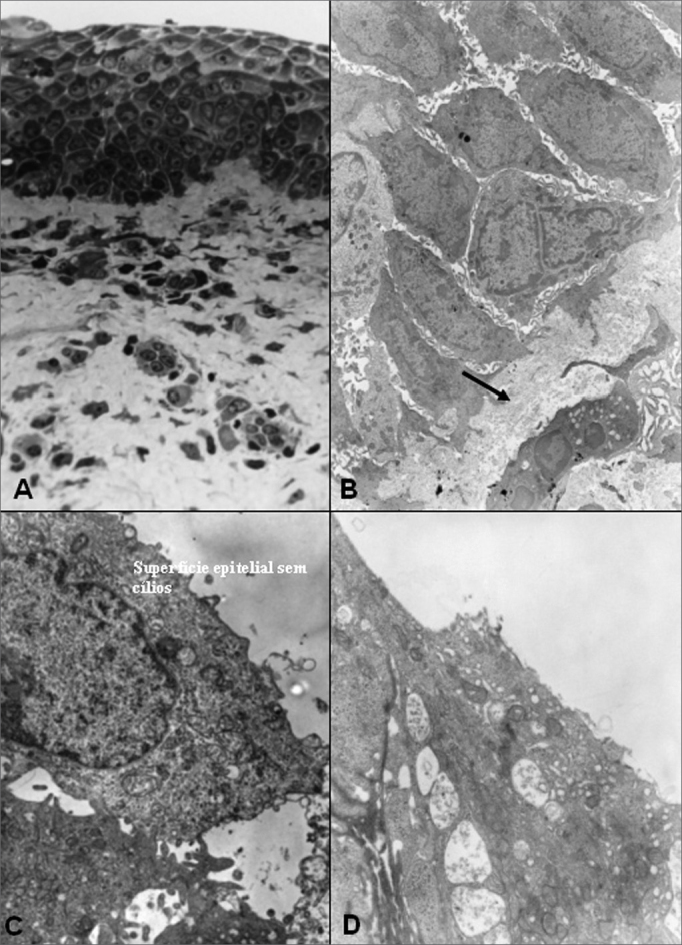
Group A - A: OM: squamous stratified epithelium (63X); B: EM: basal cells and basal membrane -MB (arrow) of stratified epithelium, with an increase of intercellular space -EI (7500X); C: EM: bold epithelial surface -absence of microvilli and cilia (20000X); D: EM: detail of superficial squamous cell (20000X).

**Figure 9:**
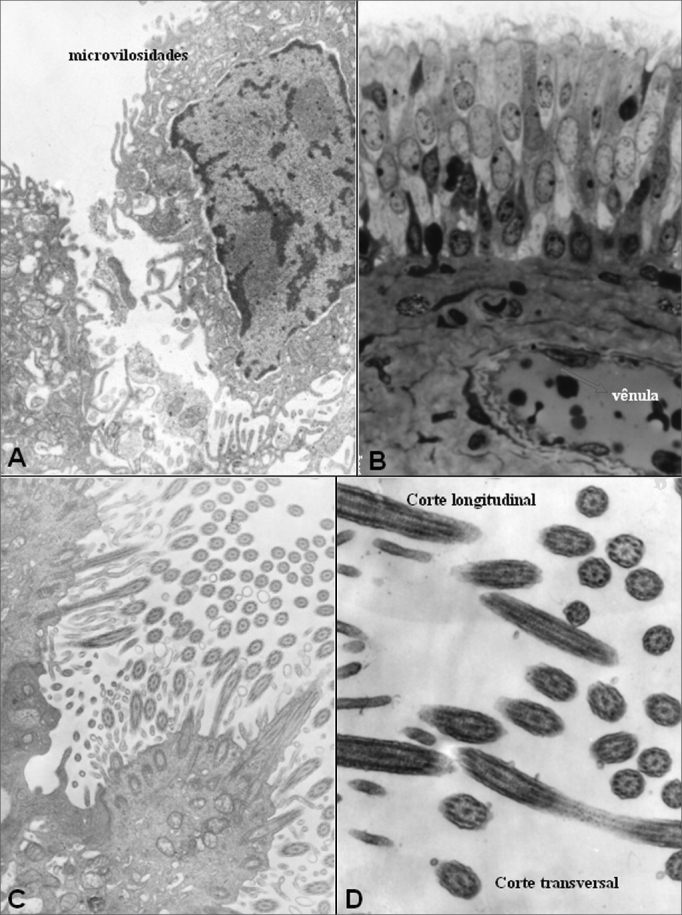
Group A - A: EM: detail of cell with microvilli and absence of cilia; B: OM: ciliary cylindrical pseudostratified epithelium. Presence of venule (arrow) in lamina propria (63X); C: EM: surface of ciliar cylindrical pseudostratified epithelium -arrows(20000X); D: EM: detail of cilium in normal cross sectional and longitudinal cuts (30000X).

The number of goblet cells observed in both groups was considered normal for the different epithelial types studied. Despite the fact that this was not the main aim of this study, some descriptions on the lamina propria were done. On the post-operative stage with the exception of one patient on group A, who mentioned the occasional use of sympathomimetic vasoconstrictor (considered non-abusive), the only nasal active medication reported by patients was nasal steroid sprays. In both groups (A and B), exactly half the patients in each group, used this type of drug, but none of them used it continuously. With regard to the two patients (both in group B), complaining of snoring, one of them reported partial snoring improvement after nasal surgery, and the other one did not think it changed much. Photomicrographs and its descriptions with the main light microscopy findings (LM) and those of electron microscopy (EM), are showed on Figures 8, 9 (group A), 10 and 11 (group B). The increase of photomicrographies corresponds to their own original increase.

## DISCUSSION

Chronic nasal obstruction represents the main reason for seeking medical help, and the most important complaint of patients, besides being also the most important target to be corrected with surgery. For most patients, nasal obstruction appeared to be severe on the pre-operative stage, since it was reported as constant in 75% of cases, besides affecting both nasal cavities in 90% of patients (groups A+ B together). Such seriousness is likely to be the important factor which led these patients to seek a specialist to relieve nasal obstruction. There was not significant difference between groups A and B, regarding nasal obstruction features on the pre-operative stage. Bambirra, in 19934, observed similar laterality incident of nasal obstruction, with 92.86% of patients having a bilateral complaint. The author also found similar reports regarding nasal obstruction duration, constantly present in 85% of cases.

Analyzing the results obtained in group A, we say that 90% evolved with total or subtotal improvement of nasal obstruction (considered a satisfactory result), and only one patient (10%) did not have post- operative alteration (improvement). In group B, the results were less satisfactory: 30% of patients reported a total or subtotal improvement, 30% partial and 40% without alteration of nasal obstruction. Despite the sample not being large enough for a wide statistical analysis, we could see evidence that PIT post-operative results for the relief of nasal obstruction is better in an earlier stage than after two years of surgery, when nasal obstruction comes back to affect a larger number of patients. It is worth mentioning that studies like this, which requires patient collaboration to come back on specific days, and mainly, the acceptance of submitting themselves to a nasal biopsy, present technical and ethical limitations towards obtaining a large sample^5^, ^6^, ^7^, ^8^.

**Figure 10:**
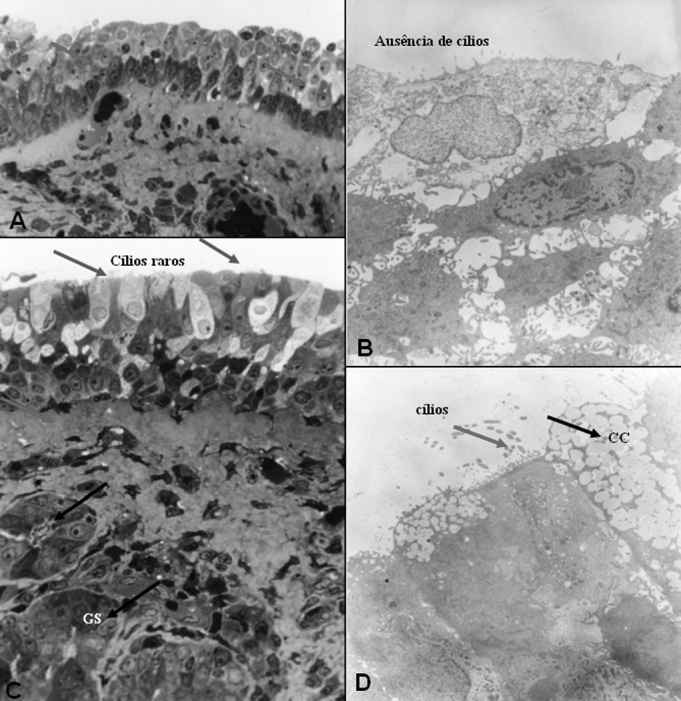
Group B - A: LM: non-ciliary cylindrical stratified epithelium, with arrows showing transition areas to cuboidal epithelium (63X). B: EM: details of non-squamous superficial cells of the squamous stratified epithelium (10000X);C: LM: cylindrical stratified epithelium with very rare cilia (green arrows). Presence of serum mucosal glands - GS on the lamina propria (black arrows) and thickness of basal membrane (red arrow). (63X). D: EM: the same patient as C, with a red arrow indicating some cilia and a black arrow on a goblet cell-CC. (7500X).

None of the patients complained of worsening in their nasal obstruction, however we should highlight that most of them already presented the same severe form on the pre-operative stage, with little or nothing to get worse.

Inferior turbinate hypertrophy was observed bilaterally on the pre-operative stage in 60% of patients (groups A and B). In group A, bilateral obstruction was present in 40% of patients, and in group B, in 80% showing a remarkable differentiation between both groups. Such findings could lead us to believe that a bilateral inferior turbinate hypertrophy has some negative influence on the post-surgical prognosis for patients in group B. In order to clarify this issue, we used the protocol assessment, where we could observe that three out of all the cases with total or subtotal improvement in group B, previously showed bilateral hypertrophy, and that, among the patients who showed unilateral hypertrophy, one showed partial improvement and another showed no alteration. Therefore, bilateral inferior turbinate hypertrophy did not appear to be an influencing variable on post-operative results.

Physical exam findings were associated to the post-operative claim on nasal obstruction. In group A, the group with the best results, only two patients showed inferior turbinate hypertrophy, one unilateral and the other one bilateral. Similarly, the worst results showed in group B, were related to a higher number of patients with hypertrophy: seven patients, three of them with unilateral hypertrophy and four with bilateral hypertrophy.

Despite being controversial, surgical treatment of inferior turbinate represents the best alternative for the relief of chronic nasal obstruction if the patient does not respond to clinical treatment. In the literature, we find several papers showing PIT efficacy, with advantages that have it at least as compatible (or better) than other current techniques for inferior turbinate surgical reduction^5^. Its easy execution and the possibility of good tissue removal are the greatest advantages, which also does not need to use larger devices or inherent costs of techniques such as laser vaporization or somnoplasty. The highest rate of scar formation and bleeding are reported as the main disadvantages for PIT^1^. However, in this study none of these findings appeared to be relevant, since we did not observe significant bleeding cases or excessive crust formation on the post-operative period. The use of oily substance associated to saline solution on the post-operative period could have contributed to less crust build up. Some authors also reported better results with PIT, in relation to other techniques such as, submucosal cauterization, cryotherapy and even the highly-praised inferior turbinoplasty^9^, ^10^.

**Figure 11:**
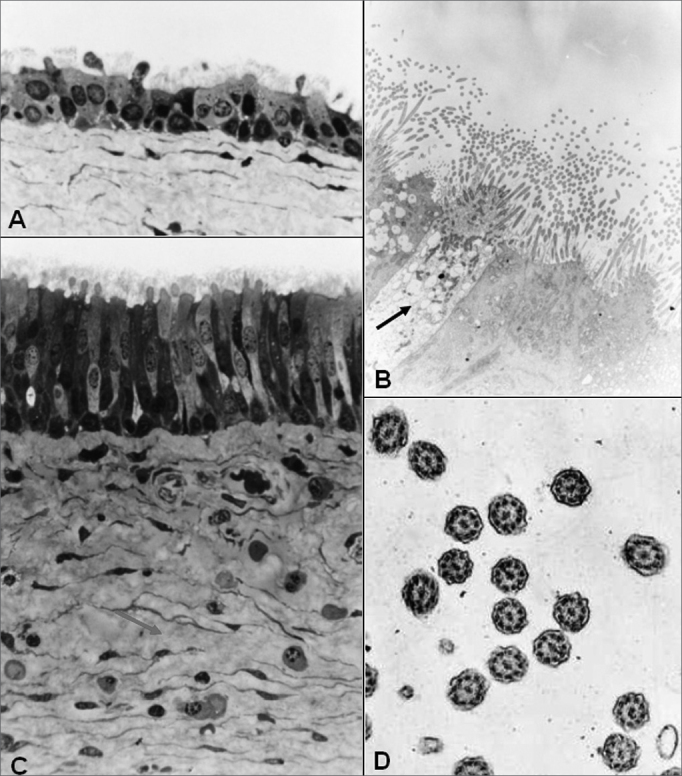
Group B - A: LM: ciliary cuboidal stratified epithelium (63X); B: MET: ciliary cylindrical pseudostratified epithelium. Arrow indicating a goblet cell (7500X); C: LM: ciliary cylindrical pseudostratified epithelium; lamina propria with loose conjunctive tissue (arrow) and cellular diversity (63X); D: MET: cross sectional cut of cillium showing its normal morphology (30000X).

In order to evaluate PIT benefits, it is a must to observe which is the post-operative stage analyzed, as it has been showed in this study, the results may vary according to the period assessed. If we analyzed patients from both groups (A+B), together, we would observe 60% total or subtotal improvement, 15% partial improvement and 25% unaltered. Results of 100% improvement in nasal obstruction in 20 patients submitted to PIT, described by Missaka (1972)^8^, referred to reassessments up to the sixth post-operative month. Such results, which are similar to those obtained for group A (90% with good evolution), may have a relation with the fact that their reassessment happened in an earlier post-operative stage.

Elwany & Harrison (1990)^9^ and Bambirra (1993)^4^ reassessed their patients one year after PIT, obtaining 75% and 82.72% improvement in nasal obstruction, respectively. Comparing these results with our study, we could say they are compatible to those results obtained in group A, because it corresponds to an approximately similar post-operative period. However, regarding group B, we found descriptions such as Meredith's (1988)^10^, who reported better results with PIT in the medium term (86% nasal obstruction improvement) than in group B (30% satisfactory improvement and 30% partial improvement), in a little bit longer period of surgical reassessment. Coutiss & Goldwyn (1990)^11^ also reported good results in longer periods of reassessment, with 72% improvement with PIT after 10-16 years (13 years average).

The reasons for better clinical results in group A when compared to group B deserve some considerations. We can imagine that a longer post-operative period, such as in group B, can be related to the time needed for the etiological factors of turbinate hypertrophy which are not controlled adequately (allergy, vasomotor factors, infections), to act causing another turbinate hypertrophy. Authors highlight the importance of continuous postoperative follow up in order to control these etiological factors^1^, ^2^. In group B reassessment, most patients had not been back to the office regularly for more than a year. Most times, according to what it was said by several patients in this group, there was an improvement of nasal obstruction on the first post-operative stage, and thus, they stopped going back to the doctor's office or even received early medical discharge. Although it has not been our goal to explain all the factors involved in hypertrophy or re-hypertrophy of inferior turbinates, we believe several factors such as: genetic, environmental, allergic, infectious, drug-related, hormonal, bio-mechanics of the nasal cavity and others, may be involved in the re-hypertrophy genesis. Anyhow, we believe it is necessary to have an extended post-operative follow up after PIT, besides having good patient education on the real benefits the surgery can offer them.

Regarding histological alterations, we observe different degrees of metaplasia, mature or not, with different types of epithelium showed^5^, ^6^, ^7^. We can find more studies with light microscopy, which described mucosal structural alterations after PIT with similar variety of epithelial types^4^, ^8^.

We did not find similar histological studies with electron microscopy after PIT, aiming to investigate ultra structural ciliary alterations. More than simply detecting the presence of ciliary epithelia on the regenerated mucosa, it was possible to determine the features of these cilia. We verified that whenever they are present, the cilia showed a completely normal ultra structure. None of the secondary ciliary alteration, reported by Jorissen (1996)^12^: composed cilia, projections or membrane loss, excess of citoplasmatic matrix, ciliary disorder, anomalies of the peripheral or central microtubules, were observed, that is to say, PIT did not appear to be a causal factor for secondary ciliary diskinesia due to ciliary ultra structural alteration. We can even observe a greater epithelial differentiation (and a smaller amount of mature metaplasia) in group B than in group A, with the presence of cilia in seven samples, four of which with ciliary cylindrical pseudostratified epithelium (breathing epithelium). This type of findings suggests that there is a total recovery of mucosa after PIT, including its ultra structural aspects, which are, according to authors such as Jorissen (1996)^12^, directly related to functional aspects of the nasal mucosa.

Thus, we verified that despite the patients in the more recent post-operative stage (group A), showing better clinical results for nasal obstruction than the later postoperative group (group B), histologically, patients form group B showed greater differentiation of structural and ultra structural elements of mucosa, with lower rates of epithelial metaplasia. According to Robbins et al. (1986)^13^, most of the times metaplasia is an undesired cellular alteration, although necessary. Besides the epithelial alterations, the lamina propria and its elements deserve being objects of other investigations in order to broaden the knowledge on hypertrophy and re-hypertrophy of nasal turbinates.

## CONCLUSIONS

1- Partial inferior turbinectomy (PIT) appeared to be an efficient surgery in relieving chronic nasal obstruction caused by hypertrophy of lower turbinates, at a short term post-operative, however, at in the medium term (after two years), its effectiveness was compromised.
2- Regenerated mucosa of inferior turbinates submitted to PIT showed possibility of total recovery of morphologic and functional components of the epithelium, showing the ciliary ultra structural as being normal.
